# Study of Crystallization Kinetics of Picromerite in the K_2_SO_4_-MgSO_4_-H_2_O System

**DOI:** 10.3390/ma19050957

**Published:** 2026-03-02

**Authors:** Songliang Ma, Yiqi Cui, Guangfeng Dong, Qingwang Liu

**Affiliations:** 1 Faculty of Land and Resources Engineering, Kunming University of Science and Technology, Wenzhi Street 34, Kunming 650093, China; 2 SDIC Xinjiang Luobupo Potash Co., Ltd., Hami 839000, China

**Keywords:** picromerite, crystallization kinetics, supersaturation, temperature, stirring

## Abstract

The crystallization kinetics of picromerite play a crucial role in optimizing the fertilizer quality. This study developed a crystallization kinetics model of picromerite. Results show that increasing temperature mainly leads to higher supersaturation, which, in turn, enhances both nucleation and growth rates, with significant improvements in crystal size and uniformity. Higher stirring speed was found to have positive effects on crystal nucleation and growth rate. The decrease in supersaturation leads to the diminution of the driving force for crystallization and the gradual decline in crystallization. The study provides a comprehensive analysis of the relationships between these crystallization conditions and the resultant crystal properties.

## 1. Introduction

As a vital component in maintaining plant health, potassium plays an indispensable role in modern agriculture [1,2]. The efficient use of potassium fertilizer is critical not only for enhancing agricultural productivity but also for ensuring global food security in the face of growing population pressures and environmental challenges. Potassium magnesium sulfate (K_2_SO_4_·MgSO_4_) fertilizer, commonly known as langbeinite, is a specialized multi-nutrient fertilizer that provides essential potassium, magnesium, and sulfur to plants [3,4]. This combination of nutrients makes it a valuable tool in modern agriculture, particularly for crops that require balanced nutrition and enhanced growth performance [5]. In addition to the comprehensive nutrient supply, potassium magnesium sulfate also has the advantage of enhanced soil health, reduced soil salinity, and environmental benefits, which makes the sulfate-based potassium fertilizers preferred over potassium chloride (KCl) fertilizer in various agricultural applications [6,7].

As a typical chlorine-free potassium fertilizer, picromerite (MgSO_4_⋅K_2_SO_4_⋅6H_2_O) is mainly produced through flotation from natural brine or chemically synthesized from potassium sulfate (K_2_SO_4_) and magnesium sulfate (MgSO_4_) through a crystallization method [8,9]. In flotation, it is well known that the particle size and morphology play a critical role in flotation processes [10,11,12]. Suitable particle sizes and shapes can significantly promote both the recovery rate and the purity of the targeted minerals [13]. Therefore, in the flotation method, if the picromerite crystal size and morphology are controlled during the natural brine evaporation process to obtain optimized particles, the subsequent flotation separation of picromerite will be more efficient. As for the chemical synthesis method, it is also essential to control the product size during the formation of picromerite crystals through the reaction of K_2_SO_4_ and MgSO_4_. The particle size of potassium sulfate fertilizer is a critical factor influencing its performance and usability [14,15]. Fine particles may dissolve too rapidly, leading to nutrient leaching and reduced effectiveness, while overly coarse particles may result in uneven distribution and suboptimal nutrient availability for plants [8]. For optimal performance, fertilizers must have a specific particle size distribution to ensure even spreading and minimize clumping [16]. As a result, precise control over the crystallization process and the regulation of the crystal properties are critical to achieving optimal product characteristics.

To attain the desired characteristics of picromerite crystals, a thorough understanding of crystallization kinetics is indispensable [17,18,19,20]. The crystallization kinetics encompasses the study of the rates of nucleation and crystal growth, which are fundamental to controlling the size and uniformity of the crystals [21]. By investigating these kinetic parameters, it is possible to develop strategies for fine-tuning the crystallization process, thereby achieving precise control over the crystal properties and improving the efficiency of the subsequent processing stages [21,22]. Research into crystallization kinetics provides valuable insights into the mechanisms governing crystal formation and growth [23,24]. This knowledge enables the optimization of crystallization conditions, such as temperature, concentration, and agitation, to produce crystals with specific attributes. Consequently, this leads to enhanced performance of the final potassium magnesium sulfate product, including better solubility, nutrient release rates, and overall effectiveness in agricultural applications.

Unfortunately, despite the critical role of crystallization kinetics in optimizing product characteristics, existing research lacks the study to fully understand the complex interplay between kinetic parameters and crystal properties. This gap limits the ability to implement precise control measures effectively, impacting the consistency and quality of the final fertilizer product. Considering that the synthesis of picromerite is a critical process in the production of high-quality fertilizers, comprehensive studies to explore the detailed dynamics of picromerite crystallization are very necessary.

Therefore, this study is dedicated to investigating the crystallization kinetics of picromerite. The purpose of the present study is to develop a crystallization kinetics model of picromerite and explore how key crystallization conditions, including temperature, stirring speed, and supersaturation, affect nucleation rates, crystal growth rates, and the morphology of the final crystal product. By elucidating these relationships, this research aims to bridge the knowledge gap and provide valuable insights for optimizing the crystallization process, ultimately enhancing the quality and performance of potassium magnesium sulfate-based fertilizers.

## 2. Materials and Methods

### 2.1. Materials

Pure K_2_SO_4_ and MgSO_4_⋅7H_2_O, purchased from Sinopharm Chemical Reagent Co., Ltd., Shanghai, China, were used to prepare pure picromerite through crystallization. Deionized water with a resistivity of 18.25 MΩ was used for all the tests.

### 2.2. Methods

Prepare a specific mass ratio of K_2_SO_4_, MgSO_4_·7H_2_O, and distilled water in accordance with the picromerite phase diagram. The mixture solution was put into a crystallizer to enable the crystallization under different temperatures and stirring speeds using a cooling crystallization method. Supersaturated solutions were prepared by first increasing the temperature by 5 °C above the target crystallization temperature to ensure complete dissolution of the salts. The solution was then cooled at a constant rate. During the cooling process, the variation in electrical conductivity was continuously recorded. When a sudden change in conductivity was observed at a certain temperature, the supernatant was immediately sampled and analyzed to obtain the corresponding supersolubility value. The supersaturation at a given temperature was calculated as the difference between the measured supersolubility and the equilibrium solubility at that temperature.

(1) Measurement of nucleation rate: Prepare a supersaturated solution of K_2_SO_4_-MgSO_4_-H_2_O, measure the volume of the saturated solution using a graduated cylinder, and then apply a probe-type online-imaging particle-size analyzer to measure the particle count during crystallization under different temperatures and stirring speeds. During the crystallization, a second chronograph is used to record the crystallization time. The nucleation rate was calculated [25] according to Equation (1):

(1) $$B_{0}=\frac{N_{total}-N}{tv},$$

where *B*_0_ is the crystal nucleation rate, *N*_total_ is the total number of particles, t is the crystallization time, and *v* is the volume of the supersaturated solution.

(2) Measurement of growth rate: In the supersaturated solution of K_2_SO_4_-MgSO_4_-H_2_O, a probe-type online-imaging particle-size analyzer was applied to measure the particle size in real time during crystallization under different temperatures and stirring speeds. The growth rate was calculated [25] according to Equation (2):

(2) $$G=\frac{l}{t},$$

where G is the crystal growth rate, l is the particle size, and t is the crystallization time.

(3) Measurement of slurry density: After measuring temperature, stirring speed, stirring time, and solution volume during the nucleation rate experiment, clean and dry the sand-core funnel and record its weight as m_0_. At regular intervals, take a sample of the solution, pour it into the sand-core funnel, and weigh it using a balance, recording the weight as m_1_. Then, dry the filtered sand-core funnel until its weight no longer changes and record this final weight as m_2_. The crystal (suspension) density was calculated using the formula [26] in Equation (3), which is as follows:

(3) $$M_{T}=\frac{{(m}_{2}-{\;m}_{0})\rho }{m_{1}-m_{0}},$$

where M_T_ is the slurry density, and ρ is the density of the mixture solutions.

(4) Measurement of supersaturation: Prepare the saturated solution and transfer it into a jacketed beaker for crystallization. At regular intervals, use a disposable syringe to withdraw a portion of the solution, filter it using a water-cooled filter head, and then transfer the filtered solution into an evaporation dish (m_1_). Weigh the dish containing the solution (m_2_). Afterward, dry the evaporation dish and weigh it again (m_3_). The supersaturation of the solution can be calculated using the following formula, Equations (4) and (5) [27]:

(4) $$C=\frac{\frac{m_{3}-m_{1}}{M_{solid}}}{\frac{m_{3}-m_{1}}{M_{solid}}+\frac{m_{2}-m_{3}}{M_{H2O}}},$$

(5) $$∆C=C-C^{*},$$

where ΔC is the supersaturation of the solution, C is the solubility of the solution at a given temperature, C^∗^ is the solubility of the solute at that temperature, M_solid_ is the molar mass of the solute, and M_H2O_ is the molar mass of water.

The crystallization process was monitored in situ using a probe-type online imaging particle shape and size analyzer (manufactured by Jinggema (Qingdao) Intelligent Technology Co., Ltd., Qingdao, China). The probe enables direct observation of picromerite crystal formation in the liquid phase, and the number of crystals increases progressively as crystallization proceeds. The instrument records particle images in real time, and the corresponding analysis software automatically identifies and counts individual crystals. Crystal number data were obtained by recording the particle counts provided by the software at predefined time intervals. The values reported in this study correspond to the raw measured data under each experimental condition. Each experiment was conducted under identical and well-controlled conditions, and the probe position and depth of vision were kept constant throughout all measurements to ensure data consistency.

## 3. Results

### 3.1. Nucleation Kinetics of Picromerite

In industrial crystallization, the nucleation rate is critically influenced by both supersaturation and slurry density, as these factors determine the number and size distribution of the resulting crystals. Supersaturation provides the thermodynamic driving force for nucleation; as supersaturation increases, the free energy barrier for nucleus formation decreases, leading to an exponential rise in nucleation rate. However, the presence of existing crystals in the slurry, quantified by slurry density, also significantly impacts nucleation kinetics. High slurry density provides additional nucleation sites due to secondary nucleation mechanisms, where existing crystals induce the formation of new nuclei through collisions or surface interactions. The combined effect of supersaturation and slurry density can be described by an extended nucleation rate expression [28], as shown in Equation (6):

(6) $$B_{0}=K_{b}{∆C}^{i}{M_{T}}^{j},$$

where B_0_ is the nucleation rate, K_b_ is a kinetic constant, ΔC is the supersaturation ratio, M_T_ is the slurry density, and i and j are empirical exponents that reflect the sensitivity of nucleation to these variables.

To fit the nucleation kinetic model of potassium salt, a supersaturated solution was prepared and crystallized under conditions of 35 °C and 800 rpm. The particle count in the solution at various time intervals was accurately measured using a probe-based in situ particle-size analyzer. The nucleation rate B_0_, supersaturation ratio ΔC, and slurry density M_T_ at different times were calculated according to the recording results, which are presented in Table 1, Table 2 and Table 3.

**Figure 1:**
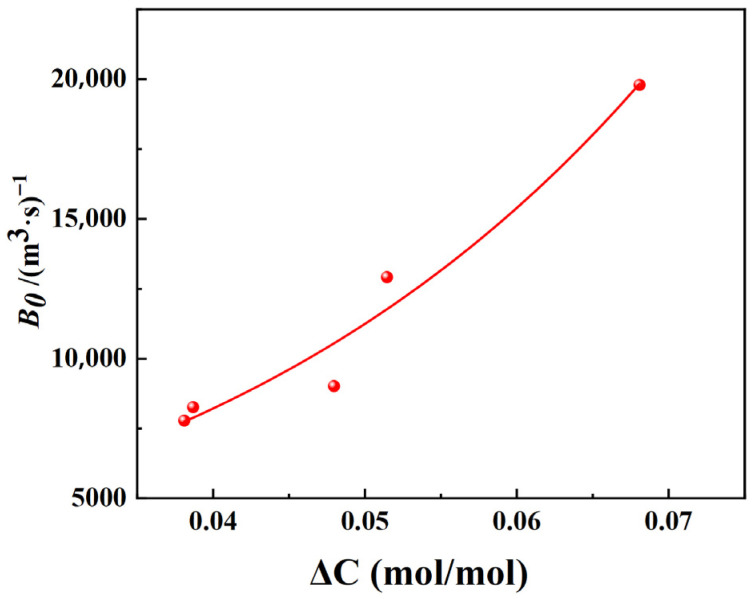
Relationship between supersaturation and nucleation rate.

**Figure 2:**
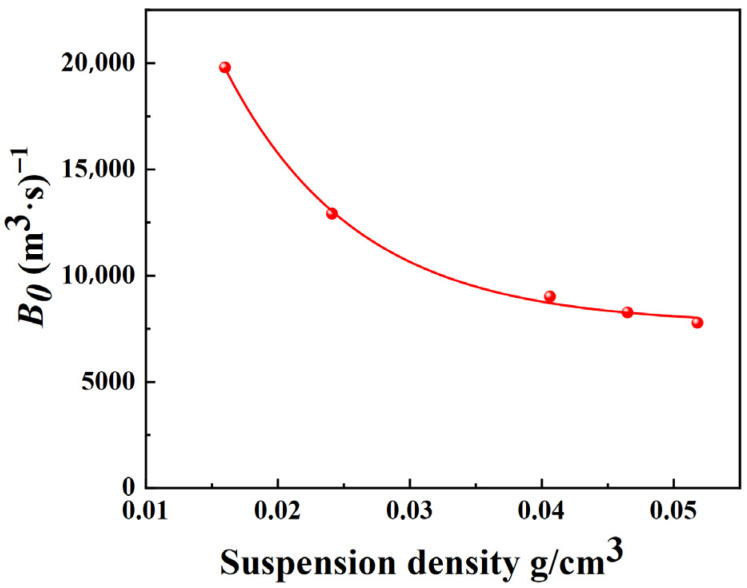
Relationship between suspension density and nucleation rate.

According to the parameters in Table 1, Table 2 and Table 3, the nucleation rates as functions of supersaturation and slurry density are plotted. According to the nucleation kinetics model from crystallization dynamics, expressed as $B_{0}=K_{b}{∆C}^{i}{M_{T}}^{j}$, a multivariate nonlinear regression was performed on the measured nucleation rates against supersaturation and crystal suspension density to obtain the parameters $K_{b}$, *i*, and *j*. The fitting results of nucleation rates with respect to supersaturation and crystal suspension density are shown in Figure 1 and Figure 2, respectively.

As shown in Figure 1, the nucleation rate of picromerite crystals increases with the rise in supersaturation. Higher supersaturation levels lead to more intense collisions between molecules in the solution, thereby facilitating the crystallization process. Figure 2 indicates that there is a negative correlation between the nucleation rate of picromerite crystals and the suspension density. As crystallization progresses, crystal particles precipitate gradually, increasing the suspension density, which in turn leads to a higher frequency of collisions between solute molecules in the solution, making it more difficult for stable nuclei to form. Furthermore, at higher suspension densities, the reduced distance between picromerite particles enhances the repulsive forces between them, increasing the likelihood of amorphous aggregation, which is unfavorable for nucleation. Additionally, a high suspension density may restrict the movement of solute molecules, making it harder for picromerite molecules to achieve the energy state required for nucleation. As a result, the increase in suspension density decreases the nucleation rate of picromerite crystals. By performing multivariate nonlinear regression on the data in Figure 1 and Figure 2, the parameters K_b_, i, and j in the nucleation kinetics model can be finally calculated; thus, the obtained nucleation kinetics model of picromerite at 35 °C and 800 r/min is determined by Equation (7):

*B*_0_ = 2.08 × 10^4^Δ*C*^0.155^*M_T_*^−0.706^. (7)

### 3.2. Growth Kinetics of Picromerite

The crystal growth kinetics model is the theoretical foundation for a deeper understanding of the crystal growth process in industrial crystallization. Growth kinetics in crystallization refers to the rate and mechanism by which crystals increase in size during the crystallization process. This phase follows nucleation and involves the addition of solute molecules or ions to the growing crystal surface. The growth rate is influenced by several factors, including supersaturation, temperature, and the characteristics of the crystal surface. The relationship between crystal growth rate G and supersaturation S can be described by the following general expression, as in Equation (8) [29]:

(8) $$G=k_{g}{∆C}^{i},$$

where G is the growth rate, k_g_ is the growth rate constant, ΔC is the supersaturation ratio, and i is the growth order.

To fit the crystal growth kinetics model of picromerite, a supersaturated solution was prepared and crystallized under conditions of 35 °C and 800 rpm. The particle size in the solution at various times was accurately measured using a probe-based in situ particle-size analyzer. The particle size l and calculated growth rate G at different times were recorded and are shown in Table 4.

**Figure 3:**
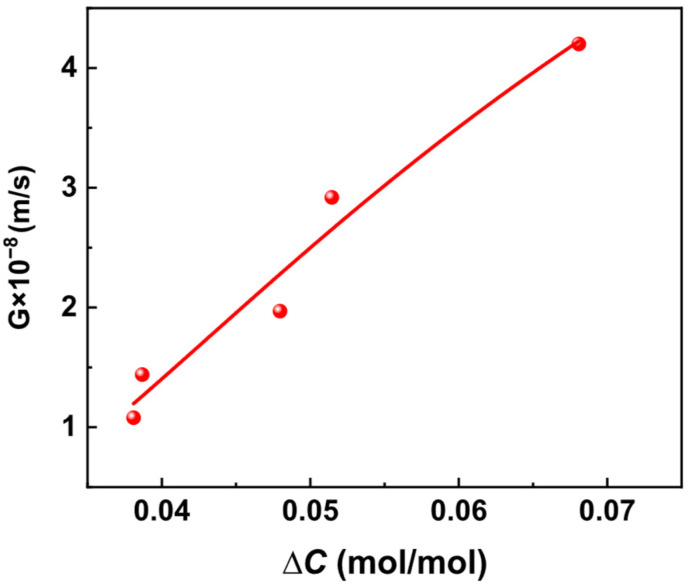
Relationship between supersaturation and growth rate.

According to the calculated growth rate G shown in Table 4, the growth rates as functions of supersaturation are plotted, which are presented in Figure 3. According to the growth kinetics model, expressed as $G=k_{g}{∆C}^{i}$, a multivariate nonlinear regression was performed on the measured growth rates against supersaturation to obtain the parameters *K_g_*, and *i*. The fitting results of nucleation rates with respect to supersaturation are shown in Figure 3.

As shown in Figure 3, with increasing solution supersaturation, the interactions between picromerite molecules or ions are enhanced. This intensified interaction leads to a greater accumulation of picromerite on the existing crystal surfaces, thereby accelerating crystal growth. Additionally, the increased supersaturation may lead to the formation of more nuclei in the solution, which serve as crucial starting points for crystal growth, further promoting the growth of crystals. Consequently, the growth rate of picromerite sulfate crystals increases with supersaturation. By performing multivariate nonlinear regression on the data in Figure 3, the parameters k_g_ and i in the growth kinetics model can be finally calculated. The obtained growth kinetics model of picromerite at 35 °C and 800 r/min is determined as follows:

*G* = 5.81 × 10^−19^Δ*C*^2.09^. (9)

### 3.3. Effect of Crystallization Time on Crystallization Kinetics

**Figure 4:**
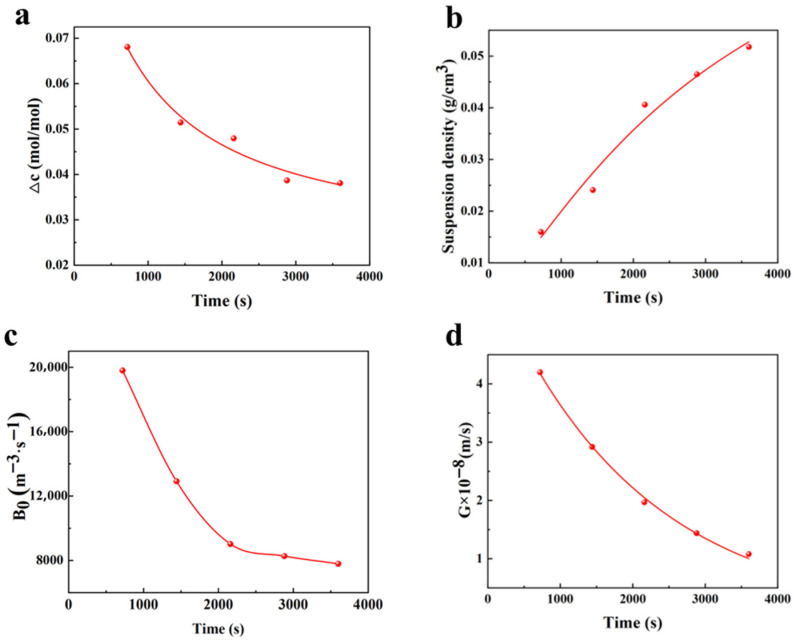
Relationship between crystallization among (**a**) supersaturation, (**b**) suspension density, (**c**) nucleation rate, and (**d**) growth rate.

Crystallization is a dynamic process, and the crystallization rate changes with the crystallization time. In this section, supersaturation and slurry density, varying with crystallization time, have been studied. As shown in Figure 4a,b, as crystallization progresses, nuclei form and grow within the solution, resulting in an increase in the number of crystal particles and a concomitant decrease in the concentration of remaining ions. Consequently, supersaturation shows a negative correlation with stirring time, while slurry density exhibits a positive correlation with stirring time. During stirring, the enhanced motion of solute molecules leads to a more homogeneous distribution of solute in the solution. This uniform distribution reduces the concentration gradient between the solution and the crystal surface, thereby diminishing the driving force for solute diffusion to the crystal surface. Additionally, prolonged stirring fosters increased collisions and interactions among solute molecules, causing some picromerite particles to reassemble into larger clusters or smaller crystals rather than contributing to the growth of larger crystals. These smaller picromerite crystals remain suspended in the solution, consuming solute and further lowering the supersaturation.

The further influence of crystallization time on nucleation and growth rates during crystallization has been investigated, and the relationship between crystallization rate and crystallization time is shown in Figure 4c,d. As crystallization progresses, the supersaturation of the solution gradually decreases, which leads to the diminution of the driving force for crystallization and the gradual decline in both the growth rate and nucleation rate of the picromerite crystals. As supersaturation progressively decreases and approaches zero, the picromerite crystals stop growing.

### 3.4. Effect of Temperature on Crystallization Kinetics

**Figure 5:**
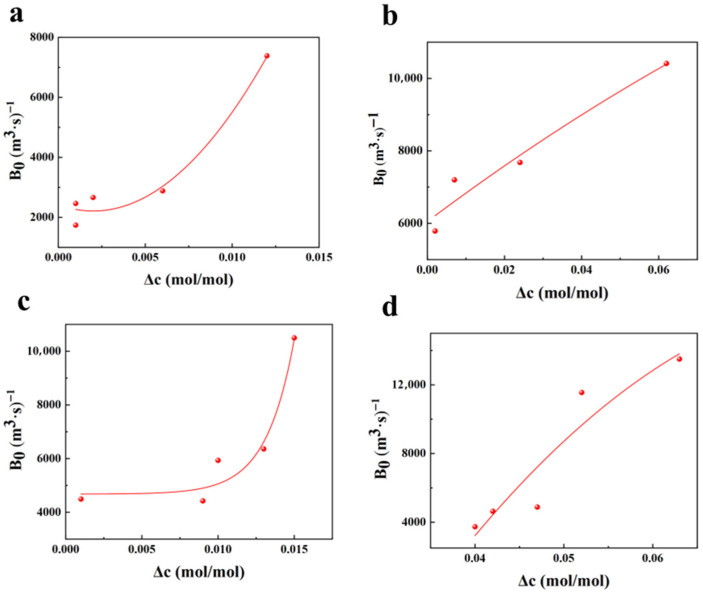
Relationship between nucleation rate and supersaturation at (**a**) 25 °C, (**b**) 35 °C, (**c**) 45 °C, (**d**) 55 °C.

**Figure 6:**
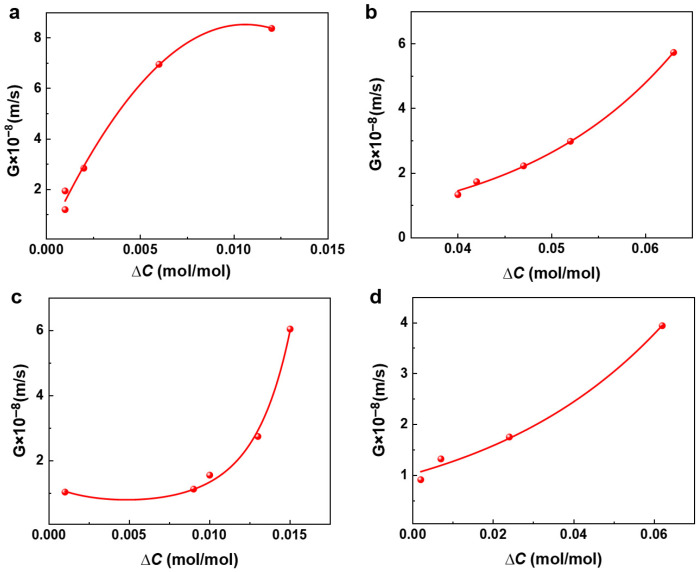
Relationship between growth rate and supersaturation at (**a**) 25 °C, (**b**) 35 °C, (**c**) 45 °C, (**d**) 55 °C.

The solubility of picromerite in solution is primarily influenced by temperature; higher temperature results in increased solubility and consequently greater supersaturation of the solution. Figure 5 and Figure 6 illustrate the relationship between nucleation and growth rates and supersaturation under different temperature conditions. The data reveal that both the nucleation and growth rates of picromerite crystals increase with increasing supersaturation. Higher temperatures enhance the solubility of picromerite, causing the solute concentration in the solution to approach its solubility limit and enter a supersaturated state. In this state, the energy required for the formation of small nucleation sites is reduced, making nucleation easier. As supersaturation rises, the concentration of solute molecules in the solution increases, creating more favorable conditions for nucleation. Consequently, both nucleation and growth rates increase with higher supersaturation. Increased temperature intensifies the thermal motion of molecules in the solution, leading to a reduction in the interactions between solute and solvent molecules. This weakening of interactions facilitates the precipitation of solute molecules from the solution to form crystals. Additionally, higher temperatures decrease the viscosity of the solution, which accelerates the diffusion rate of solute molecules. This enhanced diffusion further promotes the nucleation and growth of crystals.

**Figure 7:**
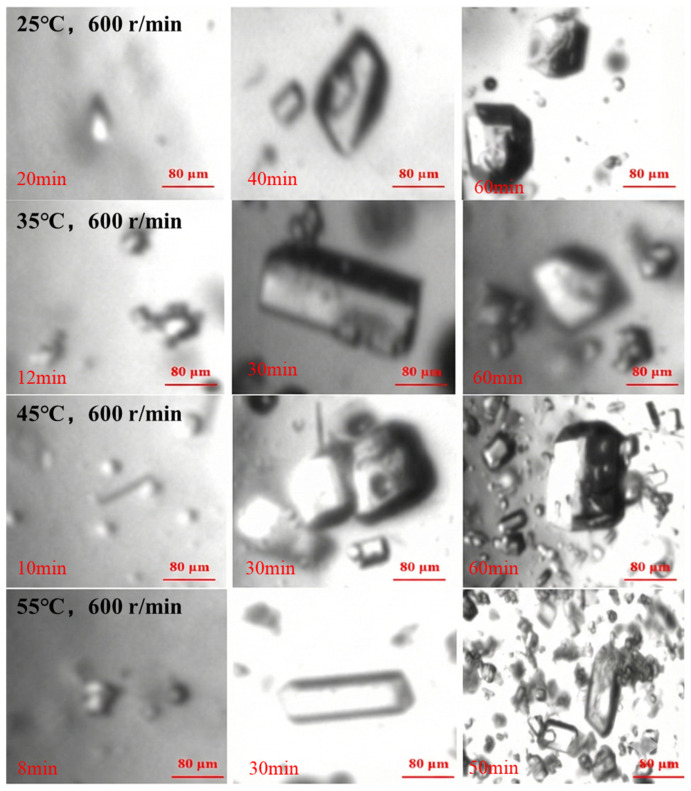
In situ monitoring of picromerite crystallization at different temperatures.

During the crystallization process, real-time monitoring of picromerite crystallization was conducted using an online-imaging particle-size analyzer. As shown in Figure 7, under higher temperature conditions, the appearance of fine crystal particles was observed to be quicker, indicating a faster nucleation rate. As crystallization progressed, the picromerite crystals rapidly aggregated and grew. For the same crystallization time, a higher solution temperature resulted in a greater quantity of picromerite crystals with a more stable particle size. The online morphology monitoring confirms that higher crystallization temperatures are more favorable for the nucleation and growth of picromerite crystals. This conclusion provides effective theoretical support for the subsequent regulation of the particle size of picromerite crystals.

### 3.5. Effect of Stirring on Crystallization Kinetics

**Figure 8:**
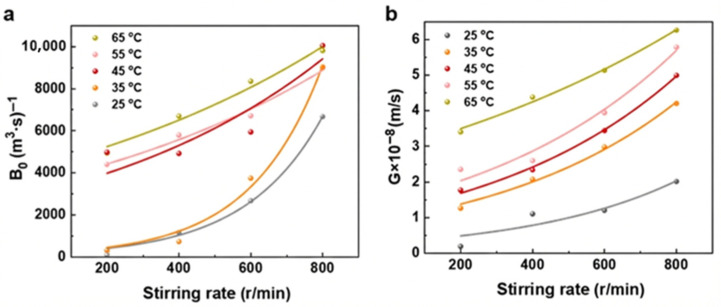
Relationship between nucleation rate (**a**), growth rate (**b**), and stirring rate.

This section investigates the effect of stirring speed on nucleation and growth rates. As illustrated in Figure 8, under the same temperature conditions, higher stirring speeds result in increased nucleation and growth rates of picromerite crystals. This can be explained by the increased mass transfer rate, enhanced probability of ion collision nucleation, and improved heat transfer rate, which facilitates heat dissipation. High-speed stirring also continuously refreshes the crystal surface, removing impurities and unreacted solute molecules adhered to the crystals, thus maintaining a clean and active surface. This surface renewal is beneficial for the adsorption and reaction of new solute molecules on the crystal surface, thereby promoting nucleation and growth. Furthermore, stirring reduces the thickness of the diffusion layer around the solute molecules on the crystal surface; a thinner diffusion layer allows solute molecules to reach the crystal surface more quickly and participate in the reaction, accelerating the nucleation and growth rates of the crystals.

**Figure 9:**
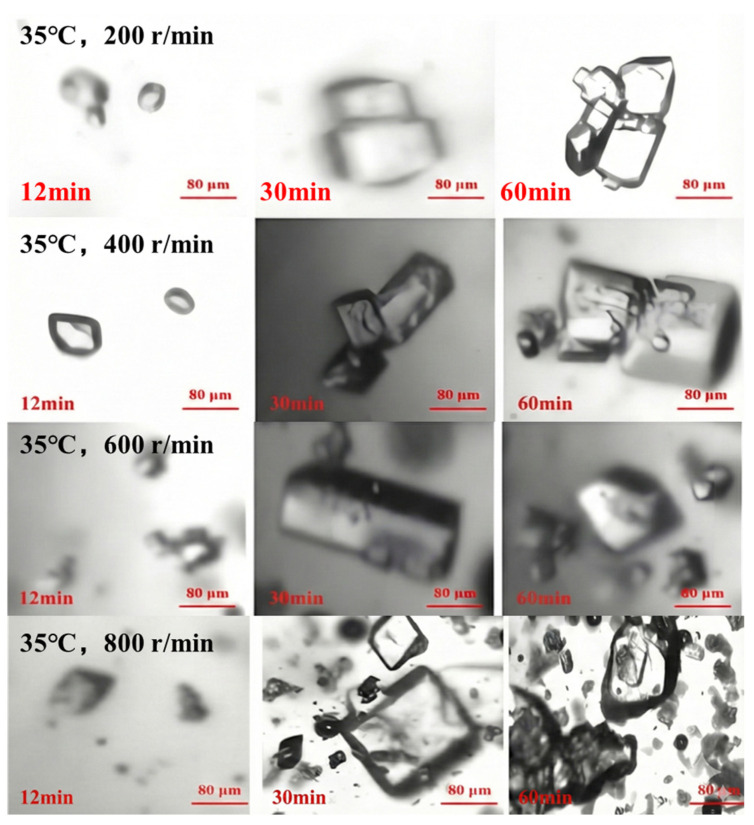
In situ monitoring of picromerite crystallization at different stirring speeds.

Real-time monitoring of the crystallization process from crystal appearance to growth was conducted using an online-imaging particle-size analyzer under different stirring speeds. As shown in Figure 9, within the same crystallization time, higher stirring speeds resulted in a larger number of picromerite crystals. Additionally, with rising stirring speeds, both the nucleation rate and the growth rate of the picromerite crystals increased, leading to a shorter crystallization process. Therefore, higher stirring speeds are more advantageous for the formation and growth of picromerite crystals.

## 4. Conclusions

In this study, the crystallization kinetic model for picromerite has been established, and the influencing factors, including crystallization time, stirring speed, and temperature, on crystallization kinetics have been studied. The main conclusions are as follows:

(1) The nucleation and growth kinetic models for picromerite crystals at 35 °C and 600 rpm are given by *B*_0_ = 2.08 × 10^4^Δ*C*^0.155^*M_T_*^−0.706^ and *G* = 5.81 × 10^−19^Δ*C*^2.09^, respectively.

(2) As crystallization progresses, the supersaturation of the solution gradually decreases, which leads to the diminution of the driving force for crystallization and the gradual decline in both the growth rate and nucleation rate of the picromerite crystals.

(3) Increasing the stirring speed and crystallization temperature leads to higher nucleation and growth rates of picromerite crystals.

## Figures and Tables

**Table 1:** B_0_ value at different times under 35 °C-800 r/min conditions.

T/s	V/m^3^	N	B_0_/(m^3^·s)^−1^
720	140 × 10^−6^	1995	19,801
1440	140 × 10^−6^	2605	12,921
2160	140 × 10^−6^	2727	9021
2880	140 × 10^−6^	3334	8271
3600	140 × 10^−6^	3926	7790

**Table 2:** M_T_ value at different times under 35 °C-800 r/min conditions.

T/s	m_2_ − m_0_ (g)	m_1_ − m_0_ (g)	ρ (g/cm^3^)	M_T_ (g/cm^3^)
720	0.023	1.7	1.19	0.0159
1440	0.037	1.7	1.11	0.0241
2160	0.049	1.4	1.16	0.0406
2880	0.073	1.9	1.20	0.0464
3600	5.368	128.5	1.24	0.0518

**Table 3:** ∆C value at different times under 35 °C-800 r/min conditions.

T/s	m_1_ (g)	m_2_ (g)	m_3_ (g)	∆C (mol/mol)
720	47.02	48.59	47.88	0.068
1440	48.06	49.04	48.52	0.051
2160	46.34	47.36	46.88	0.047
2880	48.28	49.78	48.99	0.039
3600	5.4514	6.2067	5.9197	0.038

**Table 4:** G value at different times under 35 °C-800 r/min conditions.

T/s	l/m	G/(m/s)
720	30.25 × 10^−6^	4.2 × 10^−8^
1440	42.1 × 10^−6^	2.92 × 10^−8^
2160	42.6 × 10^−6^	1.97 × 10^−8^
2880	41.42 × 10^−6^	1.44 × 10^−8^
3600	38.88 × 10^−6^	1.08 × 10^−8^

## Data Availability

The original contributions presented in this study are included in the article. Further inquiries can be directed to the corresponding authors.
